# VAReporter: variant reporter for cancer research of massive parallel sequencing

**DOI:** 10.1186/s12864-018-4468-5

**Published:** 2018-05-09

**Authors:** Po-Jung Huang, Chi-Ching Lee, Ling-Ya Chiu, Kuo-Yang Huang, Yuan-Ming Yeh, Chia-Yu Yang, Cheng-Hsun Chiu, Petrus Tang

**Affiliations:** 1 Department of Biomedical Sciences, Chang Gung University, Taoyuan, Taiwan; 2 Genomic Medicine Research Core Laboratory, Chang Gung Memorial Hospital, Linkou, Taiwan; 3 Department and Graduate Institute of Computer Science and Information Engineering, Chang Gung University, Taoyuan, Taiwan; 4 Molecular Medicine Research Center, Chang Gung University, Taoyuan, Taiwan; 5 Graduate Institute of Pathology and Parasitology, National Defense Medical Center, Taipei, Taiwan; 6 Graduate Institute of Biomedical Sciences, Chang Gung University, Taoyuan, Taiwan

**Keywords:** NGS, Exomes, SNV annotation, TCGA, ICGC

## Abstract

**Background:**

High throughput sequencing technologies have been an increasingly critical aspect of precision medicine owing to a better identification of disease targets, which contributes to improved health care cost and clinical outcomes. In particular, disease-oriented targeted enrichment sequencing is becoming a widely-accepted application for diagnostic purposes, which can interrogate known diagnostic variants as well as identify novel biomarkers from panels of entire human coding exome or disease-associated genes.

**Results:**

We introduce a workflow named VAReporter to facilitate the management of variant assessment in disease-targeted sequencing, the identification of pathogenic variants, the interpretation of biological effects and the prioritization of clinically actionable targets. State-of-art algorithms that account for mutation phenotypes are used to rank the importance of mutated genes through visual analytic strategies. We established an extensive annotation source by integrating a wide variety of biomedical databases and followed the American College of Medical Genetics and Genomics (ACMG) guidelines for interpretation and reporting of sequence variations.

**Conclusions:**

In summary, VAReporter is the first web server designed to provide a “one-stop” resource for individual’s diagnosis and large-scale cohort studies, and is freely available at http://rnd.cgu.edu.tw/vareporter.

## Background

Precision medicine based on massive parallel sequencing technologies is becoming a new trend in the treatment of diseases because it enables improved identification of disease targets, which can reduce health care costs and improve clinical outcomes. This has prompted the move of massive parallel sequencing into the clinic – the U.S. Food and Drug Administration (FDA) approved the first massive parallel sequencer in 2013 for use in clinical setting for searching known diagnostic variants in known disease genes [1]. Many massive parallel sequencing-based multiplexing assays with panels of disease genes have been developed to offer precise molecular diagnoses; these assays comprise nearly all of the Mendelian genes listed in the Online Mendelian Inheritance in Man (OMIM) database [2] and the cancer-associated genes in the Catalogue of Somatic Mutations in Cancer (COSMIC) [3], reflecting the increasing needs of and advances in genetic testing.

While most rare or novel variants are not covered by the currently available disease-targeted sequencing methods, more extensive screening approaches, such as whole-exome sequencing (WES) and whole-genome sequencing (WGS), may assure the most comprehensive collection of variant spectra from individual genomes. Recently, WES has gradually become a dominant genetic test in the diagnostic setting – it decreases the cost of sequencing and has revealed several pathogenic mutations [4–6] and medically actionable targets for subsequent therapeutic research. Despite the potential to provide comprehensive catalogues of genetic profiles, the cost, time and computing resources required to gather all of the genomic information have limited the wide adoption of the WGS assay for clinical applications [7]. Nevertheless, notable accomplishments, such as uncovering important roles of rare genetic variants in common diseases, providing deep characterization of genetic polymorphisms in different human populations, and finalizing the mutation landscapes for the most common cancer types, have still been primarily based on the use of WGS by large-scale genome sequencing centers [8, 9]. However, exploiting such large amounts of data is a substantial challenge for most researchers without bioinformatics support.

To the best of our knowledge, targeted enrichment sequencing is becoming a widely-accepted application for diagnostic purposes and is able to interrogate known diagnostic variants in addition to identifying novel disease markers from panels of entire human coding exomes or disease-associated genes. Although sequencer manufacturers have provided cloud-based solutions for general analysis purposes, these tools are bundled with specific genetic testing products from the relevant manufacturers, which substantially limits their usability. Moreover, most of the existing variant annotation tools [10–12] can perform well on single datasets and are thus suitable for clinical diagnostic tests for individuals. However, they are less likely to meet the requirements for cohort studies because cross-sample analysis is often resource demanding and not readily resolved. Here, we present VAReporter, which is web-based application with an intuitive and friendly environment for prioritizing disease-relevant abnormalities from single patients or study cohorts. VAReporter can provide comprehensive annotation by integrating a wide variety of biomedical databases. Comparison of gene mutation spectra between study cohorts and the Cancer Genome Atlas (TCGA) tumors is feasible with the aid of the visual analytic framework embedded in VAReporter. Moreover, state-of-art algorithms that account for mutation phenotypes are used to rank the importance of mutated genes. Our system also follows the American College of Medical Genetics and Genomics (ACMG) guidelines [13] for nomenclature, interpretation and reporting of sequence variations. In conclusion, VAReporter is designed to meet the requirements of massive parallel sequencing variome studies, ranging from individual diagnostic tests to large-scale cohort studies.

## Methods

### VAReporter framework

**Fig. 1:**
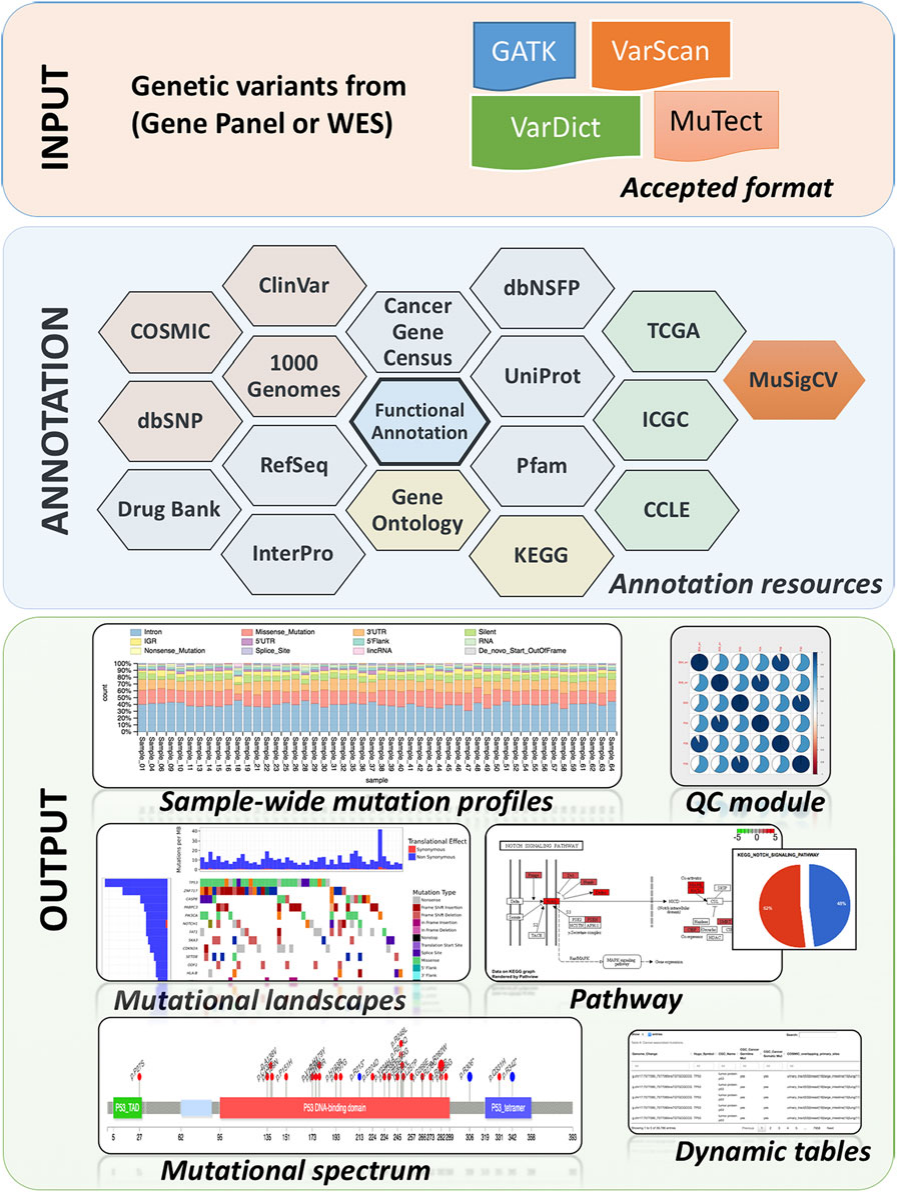
Framework of VAReporter. VAReporter takes genetic variants from gene panel or whole-exome sequencing as input materials, supporting heterogeneous VCF formats such as GATK, VarScan, MuTect and VarDict. A wide variety of biomedical databases were compiled as local annotation resources to facilitate the interpretation of biological effects introduced by genetic alterations. MutSigCV algorithm was also incorporated into the framework to detect significantly altered genes in study cohorts. Visualization modules are widely used for displaying sample-wide mutation profiles, landscapes, spectra and affected pathways. Dynamic tables with filtering and sorting functionalities are provided to facilitate the prioritization of clinically actionable targets

VAReporter provides an intuitive interface and flexible infrastructure for the management and analysis of genetic variants identified from massively parallel sequencing projects (Fig. 1). The system has functionalities that prioritize phenotype-associated variants by annotation, functional prediction, multi-sample comparison, and visual interpretation of the genetic variants. VAReporter has the ability to accept heterogeneous variant call file (VCF) formats from state-of-the-art variant callers, such as GATK [14], VarScan [15], MuTect [16] and VarDict [17], and provides the most comprehensive list of support formats with respect to single and paired samples. A wide variety of biomedical databases, including dbSNP [18], 1000 Genomes [19], COSMIC, the Cancer Gene Census [20], dbNSFP [21], Clinvar [22], OMIM [2], RefSeq [23], UniProt [24], Pfam [25], GO [26], KEGG [27], DrugBank [28], the DGIdb [29] and the Human Gene Mutation Database [30] (HGMD), were compiled as local annotation databases to facilitate the interpretation of biological effects introduced by genetic alterations. A high-performance computing cluster with Sun Grid Engine was used to fulfil the computational requirements for measuring variant accuracy and quality, annotating genetic variants, identifying significant mutated genes, and comparing mutation spectra across samples. Dynamic charts, filterable tables and reproducible reports were constructed with Shiny (https://shiny.rstudio.com), which is a web application framework for R, to facilitate data interpretation and target prioritization. For large-scale cohort studies of paired tumor-normal (T/N) samples, lists of single-nucleotide variations, insertions and deletions were subjected to the MutSigCV algorithm to identify the significantly mutated genes from WES or WGS.

### Data input

VAReporter begins by uploading a compressed file containing VCF files into the R data structure and storing the genomic features of the individual variants in a sample-specific manner. To make the subsequent quality control and annotation easier and to provide comprehensive support for the data formats of commonly used variant calling tools, such as GATK [14], VarScan [15], MuTect [16] and VarDict [17], the user can assign the corresponding data formats to the relevant VCF files via a drop-down list of variant callers before uploading the compressed file. For cohort studies, sample metadata records can also be uploaded alongside the variant files regardless of whether they are in the VCF format, the International Cancer Genome Consortium (ICGC) TSV, or TCGA MAF formats to perform in-depth comparisons between experimental designs or features. Detailed instructions on the metadata formats can be found on the tutorial page (http://rnd.cgu.edu.tw/vareporter/bookdown-tutorial/_book/intro.html). Timestamps are used as job identifiers and are returned to the user to retrieve the finished jobs.

### Quality control and association analysis

**Fig. 2:**
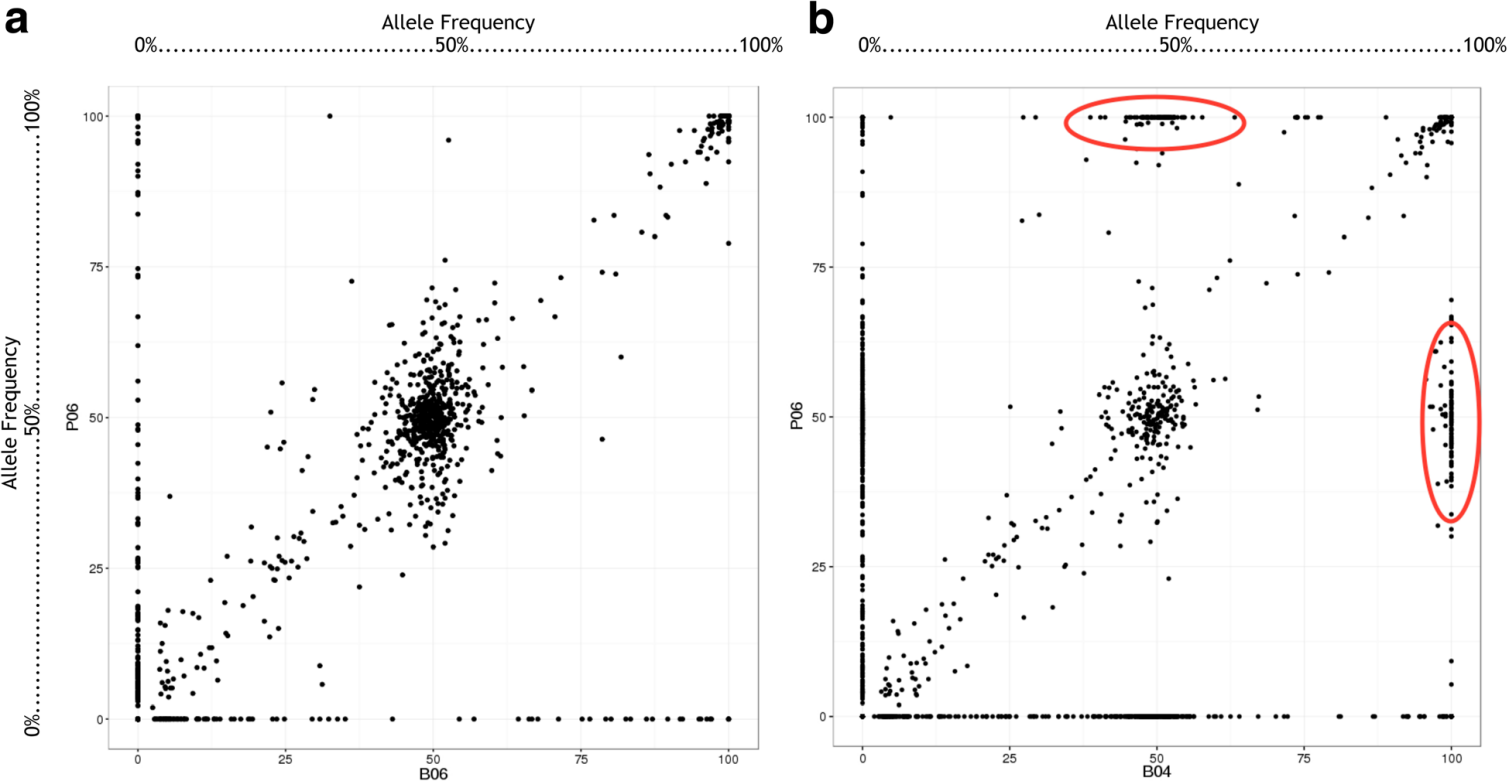
Identification of mislabeled specimens. The variant allele frequencies are extracted from T/N paired samples according to unique mutation events defined by chromosome, position, reference allele and variant allele, subsequently used to generate a scatter plot between two samples. **a** A significant portion of shared mutations from correct T/N paired samples are distributed closer to the diagonal of the scatter plot with a large majority of heterozygous and homozygous variants located at regions of 50 and 100% allele frequencies. **b** Two additional groups of points located at the top and right axis as indicated by red ovals in the scatter plot can be easily depicted when T/N mismatched samples were used to generate this figure, based on the concept that these variants are less likely to change all their variant frequencies from 50 to 100% through mutation events

The R programming language is used to retrieve the variant allele frequencies from sample pairs according to unique mutation events defined by chromosome, position, reference allele and variant allele. The correlation coefficients between the samples are rendered according to the degree of association between the variables. The R ggplot2 [31] and corrplot [32] packages are used to render the variant frequencies from multiple sample pairs into a grid layout of multiple scatter plots (Fig. 2). Generally, a significant portion of shared mutations from T/N paired samples are distributed closer to the diagonal of the scatter plot with a fair number of sample-specific variants located on the X- or Y-axes. Two additional groups of points located at the top and right axis as indicated by red ovals in Fig. 1b can be easily depicted when T/N mismatched samples were used to generate this figure, based on the concept that these variants are less likely to change all their variant frequencies from 50 to 100% through mutation events.

### Variant annotation and functional effect prediction

As mentioned in a previous study [33], the majority of the existing variant annotation tools, including ANNOVAR [10], SNPeff [11], and Variant Effect Predictor [12], were developed for general non-cancer applications and lack the functionality to automatically select the correct transcript to capture the expected variant annotations in concordance with the existing cancer sequencing studies. The transcript list can be downloaded from Broad Institute through the following link (https://www.broadinstitute.org/~lichtens/oncobeta/tx_exact_uniprot_matches.AKT1_CRLF2_FGFR1.txt) [33], which was constructed from GENCODE version 19, composed of transcripts with 100% sequence identity with UniProt records, followed by manual selection to achieve 100% annotation in concordance with MyCancerGenome [34]. These records were subsequently utilized to determine the consequences on mutations in transcripts and proteins. Functional prediction and conservation scores for coding variants can be retrieved from pre-computed results with algorithms, such as SIFT [35], PolyPhen2 [36], LRT [37], MutationTaster [38], Mutation Assessor [39], FATHMM [40], GERP++ [41] and PhyloP [42] through dbNSFP, which can ease the prioritization of variants based on the functional influences of protein alterations.

### Dynamic tables, charts and filters

The JavaScript library DataTables [43] is used to provide features such as filtering, sorting, pagination and saving the table as a PDF. Bar charts are used to present the most frequently mutated genes, highly affected protein domains and detailed variant compositions in individual samples. Flexible filters are provided based on items, such as gene symbol, genomic location, sample name, variant classification, affected protein domain, protein change, and SNVs, in specific ethnic groups in addition to disease information. A highly-integrated framework that seamlessly connects filters, tables, and charts was created with the R Shiny web application (https://shiny.rstudio.com) and is useful in both the exploratory and discovery stages for grasping the global mutational characteristics of a cohort as well as prioritizing candidate targets of interest.

### Visual summary of genetic mutations in cancer cohorts (CoMut plot)

**Fig. 3:**
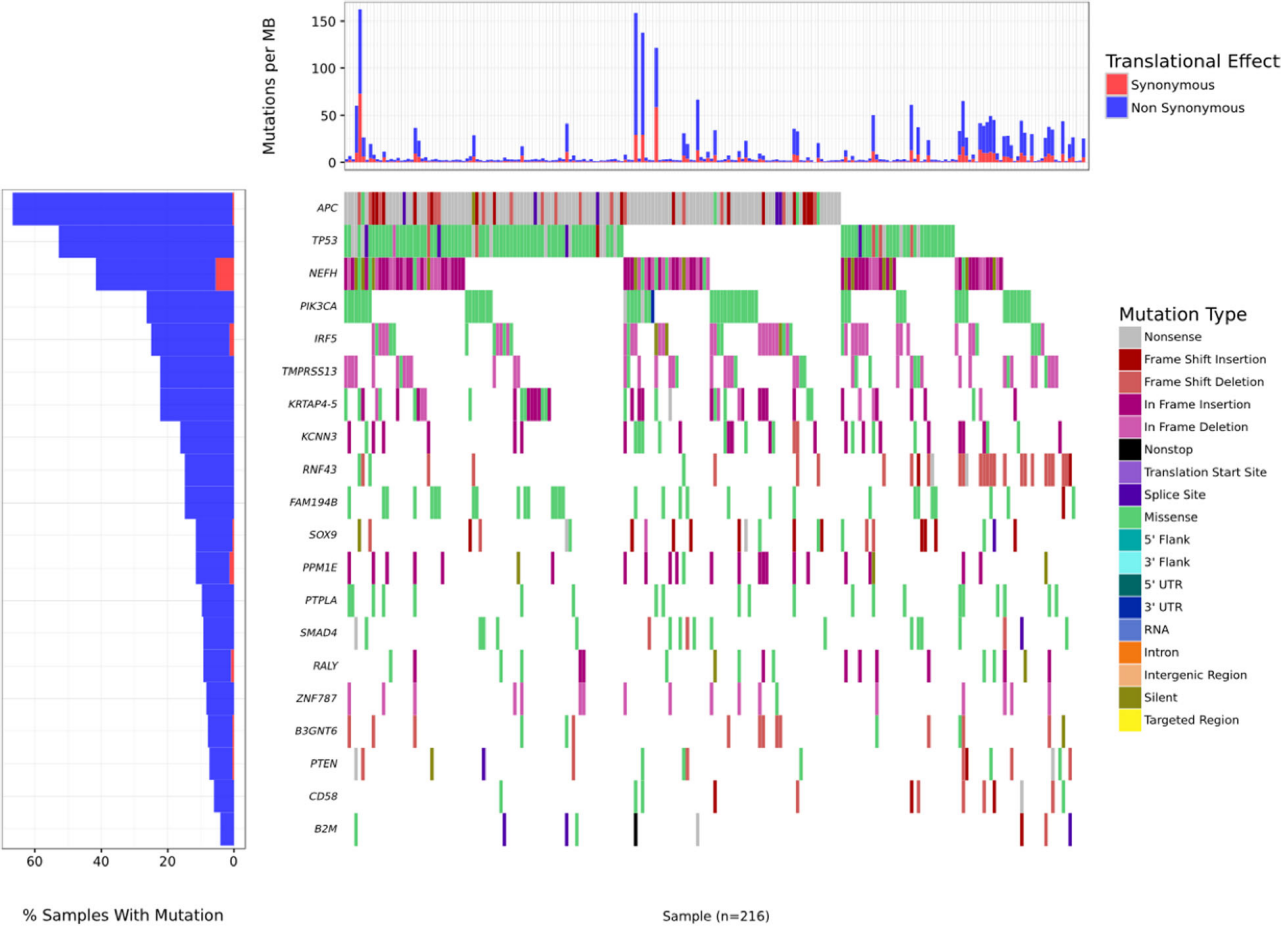
Displaying mutation landscapes by CoMut plot. In-house script is used to render significantly altered genes and their relevant mutation events into heat maps and bar graphs, which are aligned and interconnected via a common X- or Y-axes, particularly suitable for presenting data with intricate and associative natures. The OncoPrint sorting method is also adapted to display genomic alterations in the gene sets of specific signaling pathways in a mutually exclusive manner and to identify driver mutations in cancers

CoMut plots are often used in cancer research publications for visual summaries of the genetic mutations in cancer study cohorts [44]. Additionally, the MutSigCV algorithm is used to detect significantly altered genes in cancer cohorts. Because the source code for creating CoMut plots is not currently available, VAReporter uses an in-house script to render significantly altered genes into graphics similar to CoMut plots. The plots are ensembles of multiple simpler plots, such as heat maps and bar graphs, which are aligned and interconnected via common X- or Y-axes and display mutation events in a grid-like form that is particularly suitable for presenting data with intricate and associative natures (Fig. 3). Somatic genome alteration events that affect protein-coding genes within a common signaling pathway exhibit mutual exclusivity among samples, which is a well-known characteristic that is often used to identify driver mutations in cancers. To perform systematic evaluations against all signaling pathways that are plausibly perturbed by somatic mutations, the OncoPrint sorting method [45] is adapted to display genomic alterations in the gene sets of specific signaling pathways in a mutually exclusive manner.

### Comparative analysis and visualization of mutation-affected pathways

**Fig. 4:**
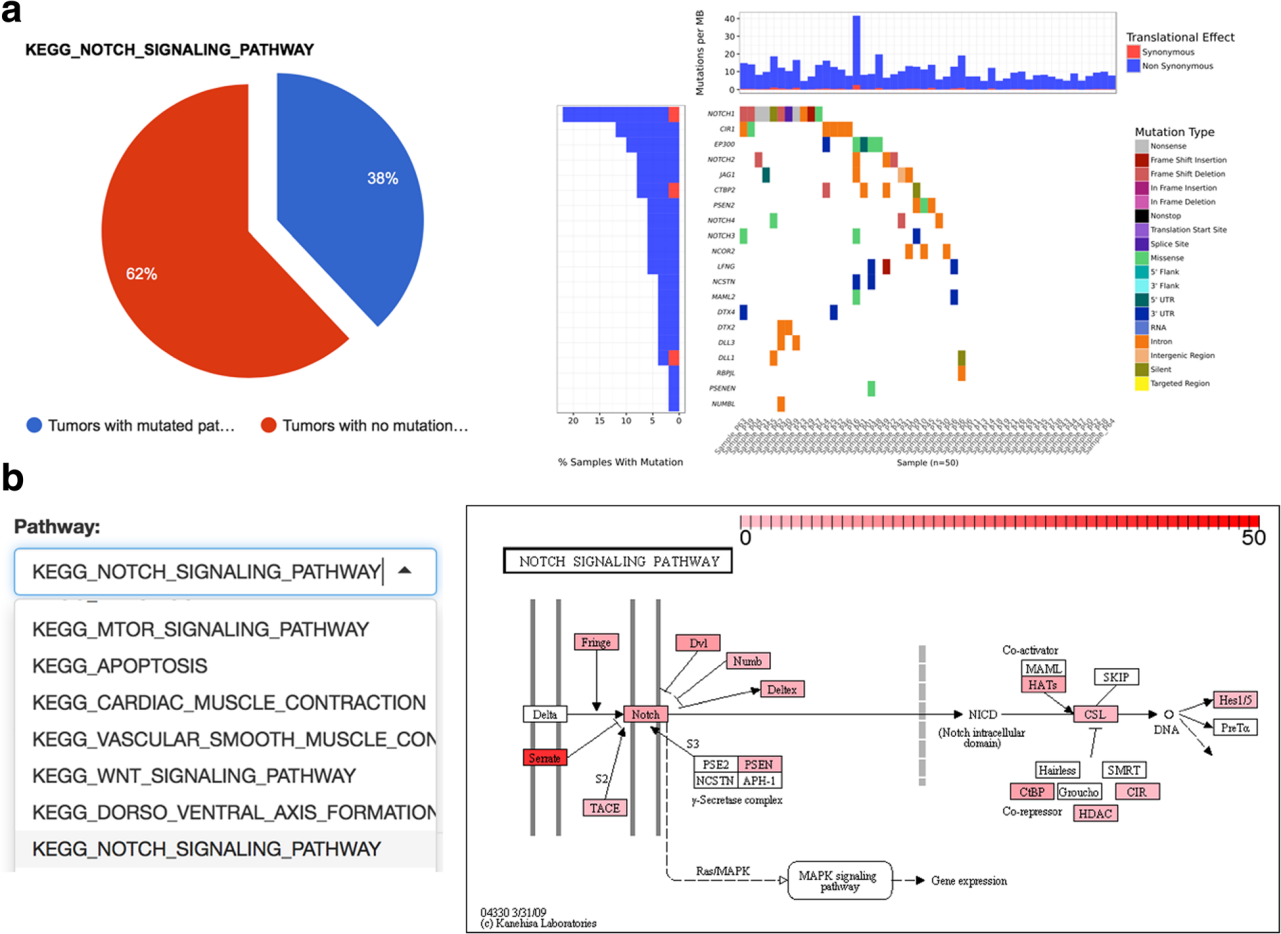
Pathway visualization. **a** VAReporter can assess the mutational events of pathway component genes and display subsets of patients as pie charts and heat maps to identify the most frequently altered pathways in a study cohort. **b** The R pathview package is used to facilitate pathway-based data integration and visualization based on mutational events identified in the component genes of specific pathway [51]

Pathway component genes are defined as gene sets collected in the Molecular Signatures Database v5.0 (MSigDB) [46] that are curated from KEGG [27], BioCarta [47], Pathway Interaction Database [48], Reactome and Signaling Gateway [49]. VAReporter can assess the mutational events of pathway component genes and display subsets of patients as pie charts and heat maps to identify the most frequently altered pathways in specific TCGA/ICGC tumors and in custom study cohorts. The GenVisR package [50] is integrated into our pipeline to facilitate the identification and visualization of mutually exclusive genetic events in pathway components (Fig. 4a). Mutational events in individual pathway component genes can be retrieved from the mutation profile, which is subsequently mapped to the relevant pathway graph downloaded from KEGG [51]. The R pathview package [52] is used to facilitate pathway-based data integration and visualization (Fig. 4b). However, only KEGG pathway maps are supported by the R pathview package.

### Comprehensive mutational spectrum analysis

Over 3 million simple somatic mutations from 66 cancer projects of the TCGA and ICGC were downloaded from the ICGC Data Portal [53] and compiled into our local index databases. Lollipop plots are a simple and widely used graphics for interpreting genetic mutations with protein annotations. An in-house script is used to translate the gene symbols into SwissProt accession numbers that can be used to retrieve protein domains and their corresponding colors from the Pfam database [25]. Diverse mutation types (e.g., missense and nonsense mutations) are denoted by different colors with marker sizes that are exponentially proportional to the number of affected samples, which provides an intuitive method for inspecting the mutational spectra in individual genes. The lollipop module can display gene-specific mutational spectra from custom cohort studies and TCGA/ICGA cancer studies at the simultaneously and side by side, which is also a unique feature of VAReporter.

### Custom gene panels and reports

As mentioned previously, VAReporter supports almost all of the available disease-targeted gene panels and whole-exome panels ranging from inherited disease genes to cancer-associated genes. Disease testing panels with known diagnostic mutations and their corresponding genes can be selected through a drop-down list provided on the web. In addition to the gene lists defined by existing commercially available gene panels, VAReporter also enables users to create gene lists for their custom-made gene panels through the panel management button.

## Results and discussion

### Example of use

As a proof-of-principle experiment, we applied VAReporter to perform an in-depth analysis of the ICGC open-access datasets, which contain 216 sets of whole-exome sequencing data from colon cancer patients in United States [54]. In this study cohort, over 796,781 mutation events that correspond to 105,739 unique somatic mutations were identified and recorded in the tab-separated TSV file, which can be downloaded from the ICGC Data Portal through the following link: (https://dcc.icgc.org/api/v1/download?fn=/release_20/Projects/COAD-US/simple_somatic_mutation.open.COAD-US.tsv.gz). A previous study [55] mentioned that calculating statistics for such huge data sets in real-time is a computationally taxing task. To address this issue, the R data structure and Shiny web framework were used to optimize the interactive visualization between the graphs and data sets. Detailed exemplary outputs for 216 mutational profiles of colon tumors can be found on the VAReporter demonstration page at (http://rnd.cgu.edu.tw/vareporter/main.php?jobId=demo.COAD-US). While this demonstration is used to profile somatic mutations, samples related to hereditary diseases may also be analyzed, from which qualitative and quantitative features of causative germline variants could be illustrated with equal efficiency. Detailed instructions can refer to the following link http://rnd.cgu.edu.tw/vareporter/bookdown-tutorial/_book/example-of-use.html .

### Output summary

After the successful submission of a job, processing statuses, such as job queuing, file format conversion, variant annotation, significant mutant gene prediction, and report generation, are displayed using a dynamic progress indicator. The output section of tutorial page (http://rnd.cgu.edu.tw/vareporter/tutorial.php) displays the standard output of VAReporter based on the demonstration data sets mentioned above. The standard output consists of eight-page sections.

### Global analyses of mutation patterns

The first section summarizes various mutation types as proportions of the total mutations per samples using a stacked bar graph to provide a global view of the mutation patterns in a sample-wide manner. Samples with relatively high or low compositions of specific mutation types can be easily depicted with this graph, which may provide some clues about the sample characteristics and their biological relevance.

The second section uses a bar chart to display the most frequently affected genes and protein domains across the samples. Basic information about each of the mutations in the affected genes and the published rankings of mutated genes for individual TCGA cancer types are also provided to facilitate comparison between the user’s data and the published results.

### Evaluating mutational consequences from the perspective of the central dogma

The third section provides a series of tables with searching, sorting, and filtering functionalities. The mutational consequences at the DNA, RNA, and protein levels are provided to enable inspection of mutations from the perspective of the central dogma. Human population genetic diversity and disease-related information [22, 30] can also be used as filters to identify ethnic-specific variants and disease-specific germline or somatic variants, respectively. For evaluating the biological consequences of novel candidate variants or mutations that have not been categorized in known variant databases [3, 18], nearly all of the available algorithms are applied to predict the importance and functional effects of each candidate variation.

### Mutational landscapes, spectra and significant mutation genes

The fourth and fifth sections employ visual analytic strategies for interactive exploration of multidimensional genomics datasets. CoMut plots are often used in TCGA cancer research publications as visual summaries of genetic variations in study cohorts. OncoPrint is a widely used strategy for identifying cancer-driver genes and pathways and can identify mutations in gene sets of specific pathways that exhibit a pattern of mutually exclusive mutations across a study cohort [56]. However, the source code for generating CoMut plot has not been released to the public and the cBioPortal constrains the OncoPrint module for use with web services only, which make their usability limited to large research institutions with well-established bioinformatics units. Although the GenVisR package [50] provides an alternative method for mimicking the functions of both CoMut plot and OncoPrint, cumbersome steps are required to annotate and render complex genomic alteration events in a cohort into the acceptable format of this visualization package. VAReporter integrates the GenVisR package and has simplified the overall data processing procedures and automated every step, including variant annotation, format conversion and CoMut plot generation. Notably, the resulting CoMut plot can be further focused on specific pathway component genes for the convenience of inspecting the mutually exclusive mutational events in individual pathways, which is a unique and novel feature of VAReporter. With the aim of identifying the dominant altered pathways in a study cohort, VAReporter can assess the mutational events of pathway component genes to identify the most frequently altered pathways in specific tumors. Lollipop plots were first introduced by cBioPortal [45] and are simple and widely used to inspect mutational spectra for individual genes and interpret genetic mutations with protein annotation. The major difference between the VAReporter lollipop module and cBioPortal is that VAReporter can simultaneously display gene-specific mutation spectra from both custom cohort studies and TCGA/ICGA cancer studies, which is also a unique feature of VAReporter. MutSigCV [57] has become a widely-accepted algorithm for distinguishing cancer driver genes from the background of random mutations and incorporates covariate factors, such as patient-specific mutation frequencies, mutation spectra, gene-specific mutation rates, gene expression levels and DNA replication timing, into the evaluation model. This design can substantially reduce false positives in the generated lists of significant genes. To simplify each data processing and preparation step, VAReporter incorporates MutSigCV [57] as a critical component for the identification of cancer driver genes. As illustrated in tutorial page (http://rnd.cgu.edu.tw/vareporter/tutorial.php), not only the significant gene list but also the summary chart of the types of genetic alterations across all samples can be created in an automatic manner.

### Mining clinically actionable drug targets

The sixth section provides tables with known information about gene-disease associations to inform clinicians of the reported mutation spectra associated with hereditary disorders or cancers. Because hundreds to thousands of coding variants can be observed in an individual’s cancer genome, prioritizing causative variants becomes a major challenge. VAReporter incorporates clinically relevant drug-gene interactions from the Drug Gene Interaction Database [29] (DGIdb) that was assembled through an extensive manual curation effort from 27 sources, including seven resources specifically focused on interactions linked to clinical trials. Users can prioritize clinically actionable drug targets by sorting scores that account for both the number of distinct sources and distinct PubMed IDs. With the potential of directly benefitting the patient, clinically actionable genes are reported alongside their drug recommendations, which may assist physicians in providing the right drug to the right patient.

### Experimental validation

The seventh section offers nucleotide sequences that span variant sites for the convenience of subsequent PCR primer design and Sanger validation. Because the Cancer Cell Line Encyclopedia (CCLE) project [58] has conducted a detailed genetic characterization of approximately 1000 human cancer cell lines, and the CCLE recorded variants are generally considered to be known mutations or verified variants, information or validation statuses on cancer-specific somatic mutations are provided to facilitate the prioritization of novel candidate mutations before experimental validations are performed.

### Identification of mislabeling errors

The final section was designed to fix the problem of mislabeled specimens in clinical labs. Specimen labeling errors account for a large proportion of identification problems during the sample collection process. Literature reviews have revealed that specimen mislabeling occurs commonly and introduces errors at rates of 0.1 to 6.5% [59]. However, existing quality assessment tools [60] only provide quality checks on sequencing data. These quality assessment methods can only provide information about the per-base sequence quality and laboratory contamination events and thus lack the ability to identify specimens with labeling errors. To provide a quick view of whether there are any problems in the data, the Pearson correlations are calculated between the samples based on the mutant allele frequencies extracted from VCF files and rendered into a correlation matrix like that displayed in Fig. 1. A scatter plot is used for the detailed inspection of variant allele frequencies between samples **(**Fig. 1**)**. The user can easily identify specimen labeling errors and incorrect T/N paired samples based on the concept that these variants are less likely to change all their frequencies from 50 to 100% through mutation events.

### Comparison of the feature of different tools for variant annotation and interpretation

Over the past few years, several packages have been developed to meet the needs of variant annotation and interpretation for massively parallel sequencing. Some tools have been designed to handle the annotation task, while other tools focus mainly on the filtering and interpretation functionalities. Despite the maturation of current tools, there still are to different extent technical weaknesses. VAReporter aims to provide the most comprehensive set of features to address all plausible issues. A more detailed comparison of the current tools is summarized in Table 1.

**Table 1:** Comparison of features of different tools for massive parallel sequencing annotation and interpretation

Tool	VARepoter	Vanno [61]	Annotate-it [55]	ANNOVAR [10]	Anntools [62]	KGGSeq [63]	SeqAnt [64]	TREAT [65]	Oncotator [33]
Availability	Web	Web	Web	Command line	Command line	Command line	Web	Command line	Both
Tracking mislabeled specimen	✓								
SNPs/1000Genomes/COSMIC	✓	✓	✓	✓	✓	✓	✓	✓	✓
Indels	✓	✓		✓	✓	✓	✓	✓	✓
Cross-sample comparison	✓	✓							
Filters	✓	✓	✓	✓		✓		✓	
Domain information	✓								✓
Dynamic summarized chart	✓	✓							
Gene Ontology	✓	✓	✓						✓
Mutational Landscape	✓								
OMIM	✓		✓					✓	✓
Pathway visualization	✓					✓		✓	✓
dbNSFP	✓	✓	✓			✓			
Sequence retrieval	✓	✓		✓					
ICGC/TCGA comparison	✓								TCGA only

### Benchmarking

The VAReporter web server runs Apache 2.2.15 on a Centos 6.8 Linux machine housing single Intel i7-5820 K 3.30 GHz processor and 64 GB RAM. To test the utility of this pipeline, 200 of variant calling files were randomly selected from WES datasets of colorectal cancer through ICGC Data Portal (https://dcc.icgc.org/api/v1/download?fn=/release_20/Projects/COAD-US/simple_somatic_mutation.open.COAD-US.tsv.gz). For the performance of VAReporter, please refer to http://rnd.cgu.edu.tw/vareporter/tutorial.php?target=benchmark.

## Conclusions

In this paper, we proposed the application of VAReporter to meet the requirements for all types of variome studies, including individual diagnostics tests and large-scale cohort studies ranging from single genes to WES/WGS. VAReporter is dedicated to the incorporation of a series of visual analytic and prediction modules for the identification of cancer driver genes, the inspection of mutational landscapes and spectra, the prioritization of clinically actionable genes, and the identification of likely mislabeled samples. Additionally, VAReporter also provides a portal for comparing in-house cancer genomic data with those from TCGA/ICGA to support comprehensive comparisons of the mutational landscapes between cohorts. Overall, VAReporter represents a highly-integrated framework for the in-depth analyses of genetic variants for all types of massively parallel sequencing applications with the aim of translating genomic data into useful clinical insights and moving toward precision medicine.

## Availability and requirements

Project name: VAReporter.

Project home page: http://rnd.cgu.edu.tw/vareporter/

Operating system(s): Platform independent.

Programming language(s): R, PHP, Shell Script and JavaScript.

Other requirements: Supported browsers Safari, Google Chrome, Firefox, Internet Explorer 11 and Microsoft Edge.

License: GNU GPL version 3.

Any restrictions to use by non-academics: none

## Abbreviations

ACMG: American College of Medical Genetics and Genomics
CCLE: Cancer cell line encyclopedia
COSMIC: Catalogue of somatic mutations in cancer
DGIdb: Drug gene interaction database
GNU: General Public License
HGMD: Human gene mutation database
ICGC: International Cancer Genome Consortium
MAF: Mutation annotation format
OMIM: Online Mendelian Inheritance in Man (OMIM) database
TCGA: The Cancer Genome Atlas
VCF: Variant call format
WES: Whole-exome sequencing
WGS: Whole-genome sequencing
